# Host-specific transcriptomic pattern of *Trichoderma virens* during interaction with maize or tomato roots

**DOI:** 10.1186/s12864-014-1208-3

**Published:** 2015-01-22

**Authors:** Maria E Morán-Diez, Naomi Trushina, Netta Li Lamdan, Lea Rosenfelder, Prasun K Mukherjee, Charles M Kenerley, Benjamin A Horwitz

**Affiliations:** Department of Plant Pathology and Microbiology, Texas A&M University, College Station, TX 77843 USA; Department of Biology, Technion – Israel Institute of Technology, Neve Shaanan Campus, Haifa, 3200000 Israel; Nuclear Agriculture and Biotechnology Division, Bhabha Atomic Research Centre, Mumbai, 400085 Mumbai, India; Present address: Bio-Protection Research Centre, Lincoln University, PO Box 84, Lincoln, 7647 New Zealand

## Abstract

**Background:**

Members of the fungal genus *Trichoderma* directly antagonize soil-borne fungal pathogens, and an increasing number of species are studied for their potential in biocontrol of plant pathogens in agriculture. Some species also colonize plant roots, promoting systemic resistance. The *Trichoderma*-root interaction is hosted by a wide range of plant species, including monocots and dicots.

**Results:**

To test the hypothesis that gene expression by the fungal partner in this beneficial interaction is modulated by the plant, *Trichoderma virens* was co-cultured with maize or tomato in a hydroponic system allowing interaction with the roots. The transcriptomes for *T. virens* alone were compared with fungus-inoculated tomato or maize roots by hybridization on microarrays of 11645 unique oligonucleotides designed from the predicted protein-coding gene models. Transcript levels of 210 genes were modulated by interaction with roots. Almost all were up-regulated. Glycoside hydrolases and transporters were highly represented among transcripts induced by co-culture with roots. Of the genes up-regulated on either or both host plants, 35 differed significantly in their expression levels between maize and tomato. Ten of these were expressed higher in the fungus in co-culture with tomato roots than with maize. Average transcript levels for these genes ranged from 1.9 fold higher on tomato than on maize to 60.9 fold for the most tomato-specific gene. The other 25 host-specific transcripts were expressed more strongly in co-culture with maize than with tomato. Average transcript levels for these genes were 2.5 to 196 fold higher on maize than on tomato.

**Conclusions:**

Based on the relevant role of *Trichoderma virens* as a biological control agent this study provides a better knowledge of its crosstalk with plants in a host-specific manner. The differentially expressed genes encode proteins belonging to several functional classes including enzymes, transporters and small secreted proteins. Among them, glycoside hydrolases and transporters are highlighted by their abundance and suggest an important factor in the metabolism of host cell walls during colonization of the outer root layers. Host-specific gene expression may contribute to the ability of *T. virens* to colonize the roots of a wide range of plant species.

**Electronic supplementary material:**

The online version of this article (doi:10.1186/s12864-014-1208-3) contains supplementary material, which is available to authorized users.

## Background

Some members of the genus *Trichoderma*, including *T. virens*, *T. harzianum*, *T. asperellum* and *T. atroviride*, are employed as biocontrol agents of plant pathogens worldwide. These soil fungi are keen mycoparasites, generally rhizosphere competent, and some have the ability to extensively colonize the outer root layers [1-4]. In addition to direct parasitism of plant pathogens, interactions with *Trichoderma* enhance plant fitness in response to biotic and abiotic stresses [5,6]. Benefits derived by the host include: a) increased plant growth [7-11], (b) increased resistance to abiotic stresses such as drought and salinity [12-16], (c) induced host defense responses to pathogens [17-23]; (d) enhanced nutrient uptake and fertilizer use efficiency [16,24-26], and (e) increased photosynthetic rates [27,28]. A recent microarray study of two dicots showed that in the plant, genes related to tolerance of oxidative and osmotic stresses are induced by the *Trichoderma*-plant interaction [29]. Growth promotion of bean plants has been found to be strain-specific [30]. The combination of close interaction with plants and the ability to tolerate heavy metals make some strains of *T. harzianum* [31] and *T. virens* [32] effective agents for soil bioremediation and plant growth promotion.

The *Trichoderma*-plant interaction has been defined as an opportunistic symbiosis [33]. The *T. asperellum* – cucumber interaction was followed by electron microscopy, and growth is extracellular, with hyphae penetrating the outer root cortex [34]. The interaction between *T. harzianum* and tomato roots was observed by confocal microscopy with a GFP-expressing strain [35]. In hydroponic cultures during early colonization (10 hours), hyphae were observed growing between plant cell walls, and by 24 h, the root surface was extensively colonized. In soil, a switch to yeast-like morphology was observed following colonization. The fungus, after 48 hours, was mainly extracellular although occasionally intracellular, and in these cases the colonized cell appears to remain viable. After longer times of interaction in soil (72 hours) the fungus produced yeast-like cells [35].

In the *T. virens* – maize interaction, GFP-expressing hyphae are observed on the root surface and growing between cell walls in the epidermis and outer cortex, with no evidence of intracellular growth [17]. The *Trichoderma*-root interaction is not identical to any other previously studied symbiosis, but the interaction is reminiscent in some ways of ectomycorrhizae (EM). Images of *Trichoderma* spp. colonizing maize [17] or tomato roots [35] show the colonizing mycelia as a loose and relatively sparse network, which is less sharply delineated than the massive EM mantle [36]. In both symbioses, mycelia penetrate the root apoplast, but it is not clear how similar are the distributions within the root. The ectomycorrhizal fungus *Laccaria bicolor* secretes a small protein (MiSSP7), highly expressed during colonization of tree roots and needed to establish the symbiosis [37,38]. MiSSP7 is imported into plant cells where it interacts with a transcriptional repressor to antagonize jasmonate-induced gene expression [39]. In arbuscular mycorrhizae, a plant nucleus-targeted effector counteracts the immune response by interacting with a specific plant transcription factor, allowing establishment of the biotrophic interaction [40]. *Piriformospora indica*, like *Trichoderma virens*, has a wide host range, interacting with roots of monocots and dicots. A recent transcriptomic study showed that this basidiomycete root endophyte tailors the expression of its genome to the host plant and to the stage of the mutualistic interaction [41].

The genomes of the cellulose degrader *T. reesei* and two mycotrophic, plant-interacting species, *T. atroviride* and *T. virens*, have been published [42,43]. The sequences have provided the tools for a genome-wide view of *Trichoderma*-fungal and *Trichoderma*-plant interactions [44,45]. Several studies have addressed the transcriptome of different *Trichoderma* species, in interaction with a particular plant host [35,46-48]. Studies of *Trichoderma* transcriptomes in interaction with plant roots, using arrays designed from *Trichoderma* ESTs, showed regulation of genes related to redox reactions, transport, lipid metabolism and detoxification [35]; small secreted and cell surface proteins, proteases, endochitinase ECH42, and novel genes that could be related to nitric oxide biosynthesis, xenobiotic detoxification, and development [46]; and a predominance of carbohydrate metabolism-genes [47]. These studies, which indicate that interaction with the plant host programs expression of many genes in the fungal partner, employed several *Trichoderma* species and times of interaction with the plant host. Here, we compared the same *Trichoderma* strain in interaction with two host plants under the same conditions, to identify host-specific transcriptomic signatures.

Although the *Trichoderma*-root interaction represents a distinct type of symbiosis, the similarities to other fungal-plant symbioses led us to the working hypotheses that (1) the fungal transcriptome should depend on the host plant species, providing a molecular basis for the wide host range; and, (2) small secreted proteins (SSPs) and other secreted proteins may be important for the mutualistic interaction and for induction of disease resistance in colonized plants. In this study we used oligonucleotide microarrays designed from the predicted protein-encoding gene models of *T. virens* [43] to ask what genes are up-regulated at the transcriptional level, comparing the interaction with either tomato or maize roots. The results of comparing two plant hosts, monocot and dicot, under the same growth conditions suggest that a different repertoire of genes is expressed in response to different hosts and provide us with a better understanding of this interaction. Such studies would be helpful for crop-specific application of *T. virens* for maximizing the benefits derived from this type of plant-microbe interactions. This study would also be helpful in isolating novel promoters for driving expression of desirable *Trichoderma* genes (e.g., elicitors) in the rhizosphere-competent species.

## Methods

### Bioassay: seedling and fungal culture

For plant interactions, maize seeds (Silver Queen hybrid) were treated with 70% ethanol for 5 min, rinsed with water, soaked in 10% hydrogen peroxide solution for 2 hours, rinsed with water, and germinated on moist sterile filter papers to screen for contamination (procedure modified from: Djonovic et al., [17]). After four to five days incubation at 27°C, five seedlings of similar root length were selected and placed on plastic screens suspended in jars containing 250 ml of half strength Murashige and Skoog (MS medium) with vitamins and 0.05% sucrose. The seedlings were grown in a temperature-controlled growth room under fluorescent light (cool white and cool daylight tubes in alternating positions, 120–150 μmol m^−2^ s^−1^) (16:8 hours, day:night) for three days at 23-25°C. Tomato seedlings were prepared by treating seeds (cultivar Moneymaker) with 70% ethanol for 20 min, rinsing with water, soaking in 10% bleach solution for 5 min, rinsing in water, and germinated on MS supplemented with 0.8% agar. After seven days, five seedlings of same root length were selected for placing in the hydroponic system.

**Figure 1:**
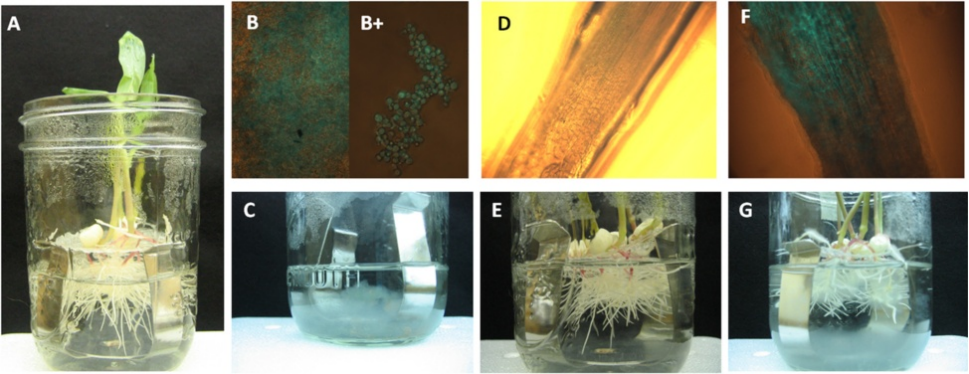
**Hydroponic system.** Experimental design for the hydroponic system for maize-*Trichoderma* co-culture at 72 h time point (similar design was used for tomato system). **A** seven-day-old maize seedlings grown aseptically in MS medium in culture chambers, inoculated with a fungal preparation of *T. virens* Gv29.8 and incubated for 72 h (See Methods). The strain expresses GFP under control of a constitutive promoter, and panels **B**, **F** show merged fluorescence and bright field images. Two controls were included: hydroponic systems containing only *T. virens* growing in MS in the absence of maize seedlings **(B, C)**, and control plants growing in MS without *T. virens* **(D, E)**. **F** and **G**, maize roots inoculated with *T. virens* at 10^5^ conidia/ml MS medium. Microscope images **B**, **D**, and **F** were taken at 10X magnification (**B**+ picture was taken with 40× magnification).

*T. virens* Gv29-8 was isolated from a sandy loam soil cultivated with cotton plants in Texas, and is deposited at the Fungal Genetics Stock Center (FGSC 10586). Conidia were harvested from 7-days old plates using a smear loop and final spore concentration was determined by dilution using a hemocytometer. Vogel’s minimal medium with 1.5% sucrose was inoculated with 1 × 10^6^ conidia/ml, and the culture was incubated at 150 rpm on a rotary shaker for 48 hours. The fungal biomass was harvested by filtering through Miracloth and rinsed with sterile water. One g of fungal tissue was added to each jar, and the seedlings (maize or tomato) were further incubated for 72 hours in the presence of the fungus. The hydroponic culture system is illustrated in Figure 1.

### Sample collection and RNA preparation

Four treatments, with three biological replicates per treatment, were collected: maize roots with *T. virens* (M + Tv); tomato roots with *T. virens* (T + Tv); maize or tomato roots without fungus (M-C or T-C, respectively); and *T. virens* without plant roots (Tv). Root systems from the same jar were harvested as an individual sample after 72 hours root-fungus interaction. Samples were gently washed with distilled water to remove clumps of fungal mycelia that adhered only loosely to the roots. The roots with remaining fungal mycelia were excised from the seedlings, frozen in liquid nitrogen, and extracted directly or stored at −80°C. Control root samples without *T. virens* were grown and harvested as described above. For axenic culture, *T. virens* was grown under the same conditions in the hydroponic culture containers, but without plants.

Total RNA was extracted from 100 mg of ground tissue (fungus and/or roots) with TRI Reagent (Molecular Research Center, Cincinnati, OH) following the manufacturer’s instructions. The quality of the RNA extracts was evaluated using the Agilent 2100 Bioanalyzer and RNA 6000 Nano kit (Agilent, Santa Clara, CA) according to the manufacturer’s instructions. Samples with electropherograms exhibiting sharp 18S and 28S rRNA peaks and showing no evidence of degradation were retained. The transcript of the *T. virens* histone H3 gene was easily detectable by RT-PCR in cDNA samples from *T. virens* and in interaction with maize or tomato, but not in control plant samples (data not shown).

### Microarray conditions and analysis

**Figure 2:**
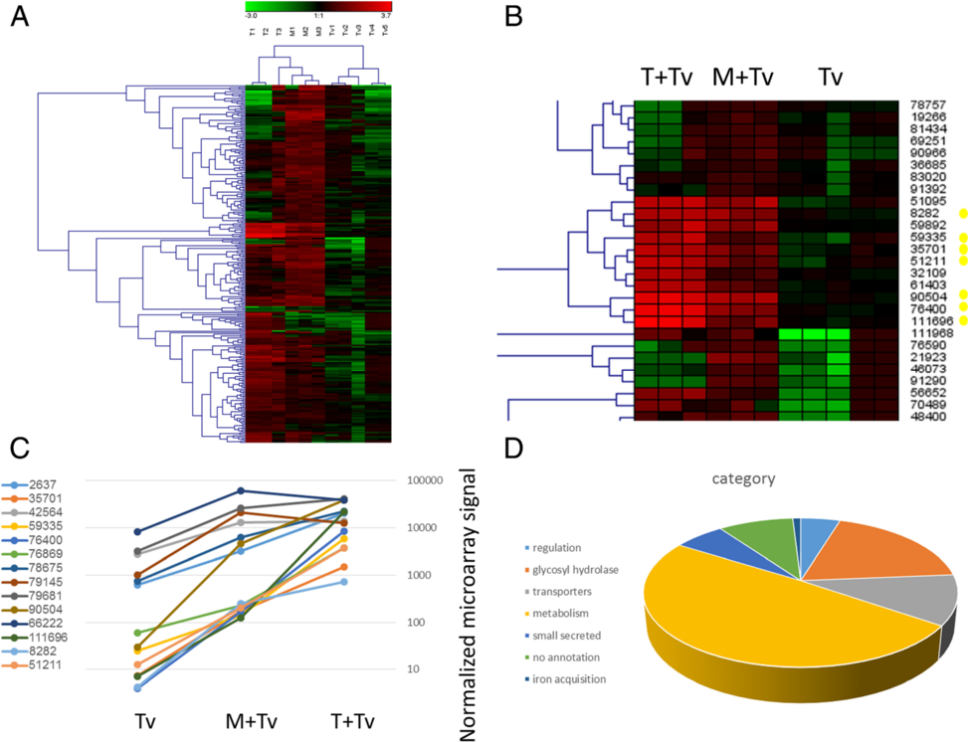
**Clustering and functional categories of significantly regulated genes. A)** Cluster analysis (Genesis, see Methods) of data from individual experiments. The log_10_ values for each microarray signal divided by the corresponding average control (Tv) signal are plotted in the heat map, clustered by relative expression level and experiment. **B)** A portion of the cluster analysis, annotated by protein ID number. This magnified portion of **(A)** shows a cluster of genes up-regulated in response to both maize and tomato roots. Yellow dots indicate 7 of the 14 genes significantly up-regulated in response to both plant hosts **(C)**. **C)** Expression patterns of genes significantly up-regulated in response to both plant hosts. Patterns are plotted for the 14 genes identified by CyberT analysis. **D)** Pie chart of functional categories for the set of genes shown in **A**. For simplicity large categories were used; the “metabolism” category includes, for example, a large number of predicted proteins with reductase, dehydrogenase, and other enzymatic activities, as well as some secreted hydrolytic enzymes with the exception of glycoside hydrolases which are listed separately. See Additional file 3: Table S2 for the complete annotated list.

The microarray platform was described in [49]. A custom microarray was designed (Genotypic, Bangalore, India) from the complete set of filtered transcript models (*Trichoderma virens* v1.0, JGI http://genome.jgi-psf.org/Trive1/Trive1.home.html [43]) and printed in 15 K arrays (Agilent, Santa Clara, CA, USA). The array consists of 12782 probes, including 536 Agilent control spots, and technical replicates for 100 probes. 11 probes had the potential for cross-hybridization; these are not among the genes of interest found in this study. The probes on the array are 60-mer sense oligonucleotides, identified by protein ID numbers from v1.0 of the database (Additional file 1: Table S4). In some cases, update of the gene models resulted in new ID numbers in v2.0. The new gene models were then identified here by BLAST search of the v2.0 database with the v1.0 sequences, and the corresponding v2.0 numbers are given in the Additional file 1: Table S4; Additional file 2: Table S1; Additional file 3: Table S2; Additional file 4: Table S3. To 1 μg total RNA, RNA was added from a spike-in kit (Agilent). Each array hybridization corresponds to a single biological replicate. In this design there are no technical replicate array hybridizations. The array contains replicated (1–10 additional spots) oligonucleotide probes, corresponding to 100 of the unique probes. Inspection of the data usually showed very similar signal values between duplicate array spots. These were analysed independently by the array software (see below). cDNA synthesis was primed with oligo dT, and the double-stranded template was used for amplification and labelling by *in vitro* transcription using the MessageAmpII kit from Ambion (Austin, TX, USA). Amplified RNA (aRNA) was labelled with Cy3 and hybridized onto the custom microarrays. Three biological replicate samples were used for M + Tv and T + Tv, and five for Tv alone. Microarray hybridization and washing steps were performed following the Agilent protocol for single-channel arrays. The arrays were scanned at 10% laser power to avoid signal saturation. Agilent’s Feature Extraction software was used to extract the data. Microarray signals were normalized to allow comparison of samples with different RNA amounts, using the spike-in data. First, the log_10_ microarray data were normalized so that signals for one of the spike-ins (E1A_r60_a20) with a log relative concentration of 3.83 had the same values across all samples. Next, the log_10_ expression data were normalized by linearly interpolating to concentrations using the ten spike-in measurements for each sample and subsequently normalizing to the 75th percentile signal intensity. The data were converted from log_10_ to linear values, and analyzed for significant differences between plant interaction and control using CyberT [50,51] http://cybert.ics.uci.edu/ with the options: Bayes window 101 and Bayes weight 8; baseline subtraction 10.0; cutoff Benjamini-Hochberg (B-H) at P < 0.05. For cluster analysis, data, as log_10_ of the microarray signal or log_10_ of the signal normalized by dividing by the average control (*T. virens* alone, Tv) signal, were processed using Genesis, http://genome.tugraz.at/ (IGB-TUG Software, Technical University of Graz, Austria, [52]) with default options for hierarchical clustering. To compare EST entries in the gene lists from [46] and [47] with our gene lists, TrichoEST ID numbers were converted to European Nucleotide Archive numbers and used to search the database (http://www.ebi.ac.uk/ena/search/). The resulting nucleotide sequences were used to search *Trichoderma virens* G29.8 v2.0 at JGI (see above), using BLASTX. The *T. virens* genomic probes in the array of [47] are identified by *T. virens* v1.0 ID numbers and directly identify probes on our array. Annotations were from the *T. virens* website (JGI). The available annotations are listed in Additional file 3: Table S2, along with a consensus description from which the functional categories in Figure 2D were determined. In addition, GO terms for the same set of significantly regulated genes (Additional file 3: Table S2) were independently identified using Blast2GO (www.blast2go.com/). The GO terms for a control set of 199 sequences, starting with protein ID 113800 (chosen arbitrarily), were analysed in the same way.

### Real time qPCR

cDNA for qPCR was synthesized starting from 2 μg of RNA, which were treated with DNase (RQ1, Promega), and then used for reverse transcription with random primers with the High Capacity cDNA Reverse Transcription Kit (Applied Biosystems), following the manufacturer’s protocol. Abundance of transcripts was measured by real-time qPCR reactions performed in an Applied Biosystems 7000 cycler; approximately 15 ng of cDNA were used as template. The 15 μl reaction volume included 7.5 μl of 2X ABsolute SYBR Green ROX MIX (ABgene, Surrey, U.K.) and 75 nM final concentration of specific primers for the gene of interest. Assays were run in duplicate for each the 4–7 biological replicate samples, using the following protocol: initial activation at 95°C for 10 min; 40 cycles of 95°C for 15 s, 60°C for 60 s, followed by a gradual increase in temperature from 60°C to 95°C during the dissociation stage. C_T_ values were calculated using the Applied Biosystems software, and transcript abundance was calculated in Microsoft Excel from C_T_ values and normalized to the tubulin (Table 1) signal. Standard curves were measured for dilution series of pooled cDNA samples, and calculated using the Applied Biosystems software; the slopes for each primer pair are given in Table 1.

**Table 1:** **Primer pairs used for qPCR amplification**

**ID v1.0**	**Consensus annotation**	**Forward primer**	**Reverse primer**	**Amplicon**	**Slope**
**90504**	Glycoside hydrolase family 7 protein	TTGGTTATGAGCTTGTGGGATGATTACTACGCCAACA	GGGAACTCCAGAGCTAGTAGAACAACTTCCTCGCTTAG	126	3.42
**46158**	Secreted short-chain dehydrogenase/reductase	GTGGAGCAGATTAGTGACACGGAAGC	CTACTGCAATACAGACCTCGACTCGCAAG	178	3.84
**86039**	Secreted NAD-dependent epimerase/dehydratase	GGTCAACGAGGAAACTCGCGCCACTAG	GTGACGGTATCATCGCGGCCAACG	182	3.37
**17705**	Small secreted cysteine rich	CAATTTTGCAAGAGTCCATTGGTTGTGTCAAC	GATTGTTGAGCTGAGTTACTGGCCGAAT	144	3.44
**19757**	Class II hydrophobin	GACTGCAAGACTCCCACTCAAGCCACCT	GGGAAGAGCATCCTGGCACAAAACAC	134	3.49
**42536**	Endoglucanase	GCTCATGATCTGGCTCGGAAAGTATGGA	GCTCATGATCTGGCTCGGAAAGTATGGAGGAGGGCGCCACAAAGCTATAAACTTG	141	3.69
**51095**	Polygalacturonase, glycoside hydrolase family 28	GACGTATCAGGTATCACTCTATCATCTATCACAGGCTATGGTA	TAGCACTGGATAGAACACCGCCGCTTCC	163	3.39
**51211**	Acetyl xylan esterase	GGATACTCACAGGGCGGCCAAATC	GTTCCGACATTATATGACAGTCCGTGAATG	175	3.87
**54057**	Secreted short-chain dehydrogenase/reductase	GCACTGCATAAGGCCCTTCAGCTAGAG	CGCCTCGCCCACGGTATCAACG	167	3.82
**56652**	Xylanase, glycoside hydrolase family 10	GCTTTACGCTTGGGATGTTGTCAATGAG	CCTTTAGTCTTGGCATAATTTGGGTCATC	191	4.25
**88010**	Beta tubulin	ACTTCAACGAGGCTTCTGGCAAC	CGGAACAGCTGGCCAAAGG	108	3.36

The slope of the calibration line is given in the last column, measured from a dilution series of pooled cDNA samples, using ABI software (see Methods). The protein model for the small secreted protein (SSP) ID 17705 is ID 230434 in v2.0 of the database.

### Reporter gene construct

We chose the CBH1 glycoside hydrolase gene (Protein ID: 90504; http://genome.jgi-psf.org/TriviGv29_8_2/TriviGv29_8_2.home.html) because the transcript is abundant in samples from root cultures inoculated with *T. virens*. Furthermore, this gene was strongly induced in interaction of *T. virens* with tomato roots in an earlier study [40]. This suggested that the upstream region includes strong expression signals that could be used to design a construct to report gene expression in response to *Trichoderma*-root interactions. A 2510 bp region upstream of the predicted translation start was amplified from *T. virens* G29.8 genomic DNA, using the forward primer P90504f, GTAGAACTGAAAAGCTTCGGTCAATC and reverse primer P90504r, CAATTTCTGATCCATGGTGTACAATCTTTG. The forward primer contains two mismatches, creating a *Hind*III site. The reverse primer contains two mismatches, converting the ATG start codon into a *Nco*I site. The product was cloned into pTZ57R/T (InsTAclone, Thermo Scientific) to obtain plasmid p90504. A 960 bp *Nco*I/*Sal*I fragment from gGFP ([53], Fungal Genetics Stock Center) containing the GFP coding sequence was ligated (T4 DNA ligase, Fermentas) to p90504, which was digested with *Nco*I and *Sal*I, and the ligation products transformed to *E. coli* HIT-DH5α (RBC Bioscience, Taiwan). Five μg of the resulting plasmid, p90504-GFP, were co-transformed together with 5 μg of pUCATPH (containing the hygromycin phosphotransferase *hph* gene with fungal expression signals [54]) to protoplasts of *T. virens* G29.8, using the PEG protocol as described by [55]. After regeneration overnight, transformation plates were overlayed with 600 μg/ml hygromycin B (A.G. Scientific, San Diego, CA) in 1% agar (Difco). Colonies reaching the surface of the overlay were transferred to PDA (Difco) with 100 μg/ml hygromycin B. About 40 hygromycin-resistant colonies were screened by inoculating wells containing excised maize root sections in 0.5x MS medium with 0.05% sucrose. After three days incubation, the wells were observed under a binocular fluorescence microscope (Olympus). Three isolates that showed GFP expression were identified and one, designated C10, was used for experiments.

## Results and discussion

### Microarray analysis of *Trichoderma*-root interactions

**Figure 3:**
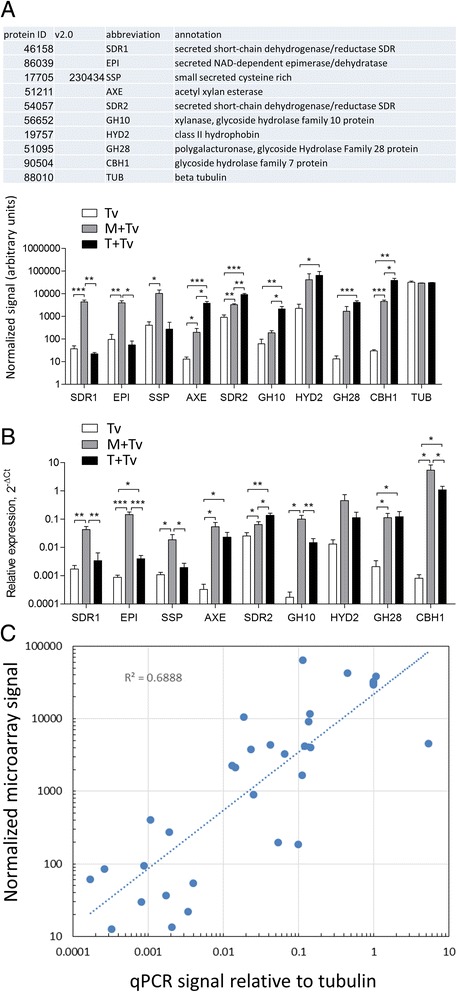
**qPCR validation of microarray data. A)** qPCR signals normalized to tubulin (ID 88010), and corresponding microarray data, normalized to total signal as described in Methods. Bars indicate means of 3–8 replicates with standard error; the M + Tv and T + Tv samples for qPCR were independent of those used for the microarrays. *,**,** significant at P < 0.05, 0.01, 0.001 respectively, by ttest, one-tailed. **B)** Microarray data corresponding to the transcripts shown in **(A)**. *,**,** significant at P < 0.05, 0.01, 0.001 respectively, by ttest, one-tailed. **C)** Correlation between qPCR and microarray signals, plotted from the data in (**A** and **B**). The line is a power-law least squares fit (Excel) to the combined data set (Tv, M + Tv, T + Tv), which appears linear on this log-log plot. The R^2^ value is shown on the graph.

**Figure 4:**
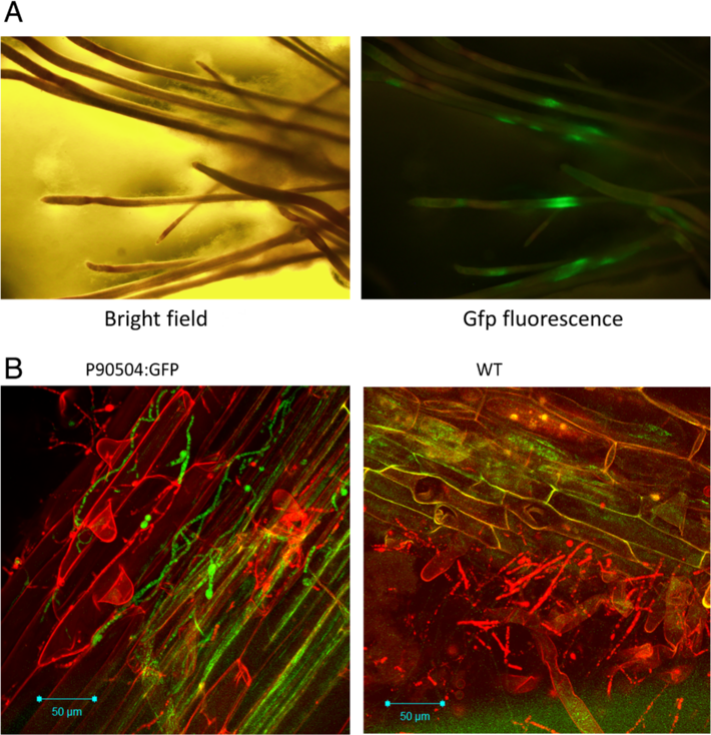
**Expression of GFP under control of the upstream region from glycoside hydrolase gene 90504. A)** Roots excised from hydroponic co-cultures of P90504:GFP line C10 were photographed under white light or for GFP fluorescence, in a binocular microscope. **B)** Confocal images of roots from hydroponic co-cultures of P90504:GFP line C10 or WT. Green channel: GFP fluorescence, excitation 488 nm, bandpass filter 500–550 nm. Red channel: propidium iodide, excitation 561 nm, filter 575 nm long pass. The images are projections of five (left) or 8 (right) Z-stack (1.5 μm) slices. Propidium iodide stains plant cell walls in the root epidermis and outer cortex layers, as well as some plant and fungal nuclei in some cells. Plant cell autofluorescence (green channel) is visible in some areas.

The hydroponic culture system used to follow *Trichoderma*-root interactions is illustrated in Figure 1. At the time of harvesting, adhering fungal mycelia were clearly visible on the roots of both plant hosts, as shown for maize in Figure 1G. For comparison of the fungal transcriptome in interaction with roots to that of *T. virens* alone, the fungus was cultured under the same conditions but without plants (Figure 1C). Total RNA for preparation of probes for microarray hybridization was prepared from material from each treatment: *T. virens* alone (Tv, 5 experiments), in interaction with maize (M + Tv, 3 experiments) and with tomato (T + Tv, 3 experiments). Complete microarray data are provided in Additional file 2: Table S1 and are available at GEO, http://www.ncbi.nlm.nih.gov/geo/query/acc.cgi?acc=GSE64344. The data sets were analyzed in pairs using CyberT (see Methods) for significant differences in transcript abundance: M + Tv compared to Tv, T + Tv compared to Tv, and M + Tv compared to T + Tv. At a Benjamini-Hochberg cutoff value of P < 0.05, the levels of 139 transcripts were significantly up-regulated in interaction with maize, and 85 in interaction with tomato (Additional file 3: Table S2). A panel of 10 genes, highlighted in the protein ID list in Additional file 3: Table S2, was chosen for qPCR validation. The primer pairs used are given in Table 1. Beta tubulin (protein ID 88010) was the reference gene for qPCR (TUB, Figure 3A). The expression patterns of these genes measured by qPCR or microarray hybridization were generally similar (Figure 3). An exception is 56652 (xylanase, GH12) where the *T. virens* control signal was underestimated by qPCR compared to microarray hybridization. The calibration slope for this primer pair indicates below-optimal efficiency (Table 1), perhaps accounting for the discrepancy. In general, the correlation between qPCR and microarray signals holds well, over several orders of magnitude (Figure 3C). The other exception is GH12 (ID 42536, Table 1) which showed no induction in interaction with maize on the microarray, as well as in one set of qPCR experiments. This transcript, however, was strongly up-regulated in a second set of qPCR experiments (data not shown). This is in contrast to the other 9 genes in the panel which showed remarkably consistent patterns in experiments performed in two different laboratories and at different times during this study. Cluster analysis was performed, according to experiment and gene name, with the group of transcript levels showing significant regulation in either root interaction (see Methods). The significantly regulated genes cluster according to experiment (Figure 2A), but some genes deviate from the pattern in some experiments (most noticeably, but not only, a cluster where tomato replicate T3 differs from replicates T1 and T2). The sources of this variation might be sampling effects (note the non-uniform expression of GFP in Figure 4A, described in detail below) or differences between hydroponic chambers in the time course of the *Trichoderma*-root interaction. As for ID 42536 (Figure 3A), time course experiments on specific genes or the entire transcriptome may be needed to resolve these differences. Thus the number of significantly regulated genes may be an underestimate, and we limit the analysis to robustly regulated transcripts.

### Genes regulated in interaction with both plant hosts

The expression of 14 *T. virens* genes was significantly up-regulated in interaction with both maize and tomato. Of these, 8 were found previously, in interaction with tomato ([47], coded by violet shading in Additional file 3: Table S2). A cluster of genes co-regulated in interaction with both host plants is shown in Figure 2B, annotated by JGI protein model ID number. Seven expression patterns of genes belonging to the set significantly up-regulated in both root interactions (Figure 2C) cluster together (indicated by yellow dots in Figure 2B). The 14 genes significantly up-regulated in interaction with both maize and tomato are mostly found on different scaffolds. In the two cases where a pair of genes was found on the same scaffold, the distance between them was very large (more than 600 kb). Similar expression patterns in the three conditions (Tv, M + Tv and T + Tv), as evident in Figure 2C, is therefore not reflected by co-localization in the genome. Ten of the 14 genes in this list are annotated as glycoside hydrolases. Seven of the eight genes found to be up-regulated by interaction with both maize and tomato in this study (sampled at 72 h) and with tomato (sampled at 20 h, [47] are annotated as glycoside hydrolases. The exception is ID 180068, encoding a protein with Zn finger and F-box domains, which might be have a regulatory function. Identification of glycoside hydrolase-encoding genes in experiments performed in different labs, and at a different sampling time, points to a core function: the ability to (partially) degrade the root cell walls.

### Genes up-regulated in interaction with either or both plant hosts

Looking at the available annotations for the larger list of genes significantly up-regulated in interaction with either plant host, glycoside hydrolases are again prominent (Additional file 3: Table S2). Another class includes transporters (Additional file 3: Table S2, Figure 2D). There are several predicted transcription factors and other regulatory proteins, and some small secreted proteins. Two genes are related to iron acquisition: Fe permease FTR1 (ID 24347, v1.0; 195287, v2.0) and a ferric reductase (ID v1.0 11584, v2.0 147314). A similar picture, in general, was independently obtained by GO term analysis (Additional file 5: Figure S1). The highest-represented biological processes are polysaccharide catabolism, transmembrane transport, and oxidation-reduction (Additional file 5: Figure S1). An identical GO term analysis using a randomly chosen set of sequences (control, Additional file 5: Figure S1) resulted in a much more diverse representation of biological processes. Oxidation-reduction is also highly represented in the list obtained from the consensus annotation (see individual annotations in Additional file 3: Table S2), although this is not obvious in Figure 2D where oxidation-reduction is included in “metabolism”.

**Figure 5:**
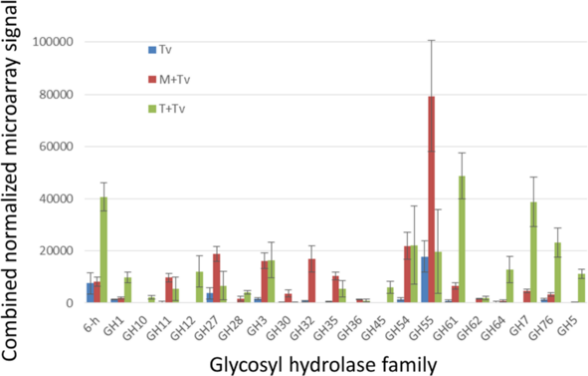
**Representation of glycoside hydrolase (GH) families.** The bars represent the sum of average transcript abundance (microarray signal) for each family. The error bars indicate SEM for families with a single member represented; for families with two or more members, the SEM values were combined using the sum of squares rule, to provide an approximate measure of variability.

Of the fourteen genes demonstrating significant up-regulation in response to both maize and tomato roots, nine are annotated as glycoside hydrolases. All the glycosyl hydrolases (a total of 34) were grouped according to GH families from the annotation (JGI), and the total expression (microarray signal summed over all the family members for each GH family) plotted to obtain a semi-quantitative view of the relative contribution of each family, at the transcript level (Figure 5). The plot indicates that the expression pattern is different for the interaction with maize or tomato.

Different cell wall composition of the two plant hosts might be expected to play a role in the set of fungal genes expressed. The predicted substrates of the fungal glycoside hydrolase families represented (following [56] and the CAZy database, http://www.cazy.org/Glycoside-Hydrolases.html) do not appear, however, to be related in any direct way to the composition of maize vs tomato cell walls. As an example, we note that dicot walls are more pectin-rich than those of monocots [57]. The endopolygalacturonase encoded by transcript 51095 is the unique member of family GH28 expressed in response to interaction with roots. If the product of 51095 is indeed the major induced pectin-degrading enzyme, one might have expected higher expression in tomato where the substrate is more abundant. This pattern was found [58] upon comparison of two *Colletotrichum* species. One species is a pathogen of Brassicaceae, and one of maize. *C. higginsianum* in the necrotrophic phase of its interaction with *Arabidopsis* was found to up-regulate a greater number of pectin-degrading enzyme genes than *C. graminicola* on maize [58]. Our data show, however, up-regulation of the GH28 gene 51095 in interaction with both tomato and maize (Figure 3A, B). Dicot and grass cell walls both contain pectin, and the expression of enzyme genes will not always correspond directly with substrate levels. The predicted ortholog (E value 0.0, BLASTP) of 51095 in *T. harzianum* is ThPG1. Silencing of ThPG1 in *T. harzianum* showed that it is required for colonization of *Arabidopsis* roots [59]. This could be tested for *T. virens* on tomato and maize. The question of how GH family expression might be related to host cell wall composition will need to be addressed at the protein, in addition to transcript, level. When and where each CAZyme is expressed in the root tissue is also important.

### Genes differentially regulated in interaction with maize or tomato

**Figure 6:**
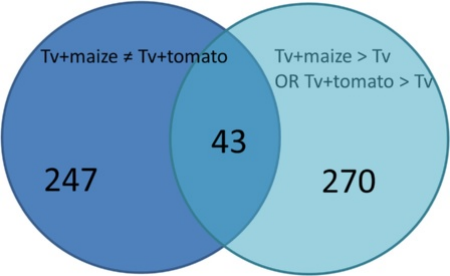
**Venn diagram of the number of genes differentially expressed in** ***T. virens*** **interactions with maize** ***vs*** **tomato roots.** The intersection of the two sets indicates transcripts that were significantly different (B-H P < 0.05 by CyberT) in tomato compared to maize, and also significantly up-regulated compared to control (Tv) in either tomato (T) or maize (M) interactions.

**Figure 7:**
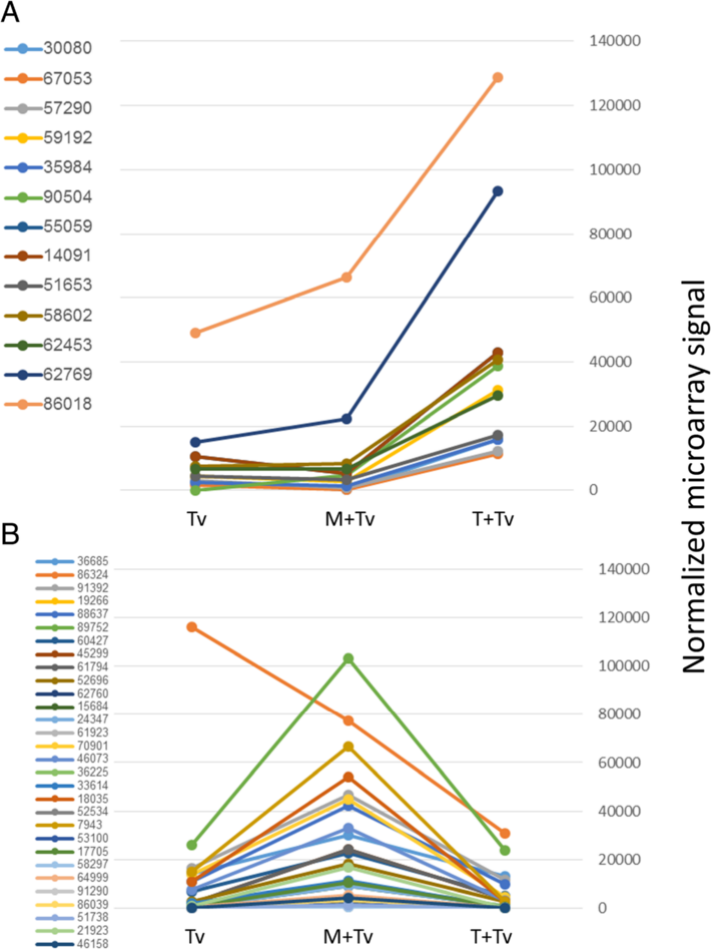
**Expression patterns of root-regulated** ***T. virens*** **genes that are preferentially expressed in interaction with tomato (A) or maize (B).**

The overall distribution of gene classes in the list of up-regulated genes is similar for the two plant hosts (Additional file 3: Table S2). Inspection of the microarray data showed that expression in the maize and tomato interactions often followed the same pattern, yet did not pass the significance filter in one or the other data set. We therefore studied the set of genes whose transcript levels differ significantly at P < 0.05 between maize and tomato, and are also significantly regulated relative to the *T. virens* control. This gene list, which is the intersection of the CyberT results from the above two criteria (Figure 6), is given in Additional file 4: Table S3. Transcripts corresponding to 43 gene models differed significantly in their expression levels between maize and tomato and also between the control and at least one plant interaction, with ratios of transcript abundance on tomato *vs* on maize ranging from 60.9 to 0.005 (Additional file 4: Table S3 and Figure 7). Thus, we have identified genes that can serve as specific reporters for the interaction of *T. virens* with one of the two plant hosts. In contrast to the genes significantly up-regulated in interaction with both hosts, of which half had been found previously, only five of the transcripts in Additional file 4: Table S3 (violet or blue color-coded) had been identified previously in the *T. virens* – tomato interaction [47] or are homologs of genes up-regulated in the *T. harzianum* – tomato interaction [46].

Maize-specific expression of three genes, oxidoreductases 46185, 86039 and SSP 17705, was confirmed by qPCR (Figure 3). The intracellular invertase TvInv (ID v1.0 21923, v2.0 111987) [27] is specific to the maize interaction, though obviously one cannot exclude induction on tomato at a different time point from the 72 h sampled here. The tomato-specific up-regulated transcripts (Additional file 4: Table S3) remain to be further characterized.

**Figure 8:**
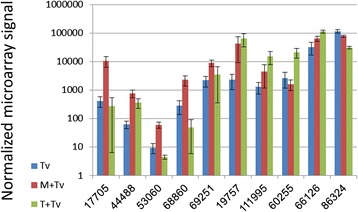
**Expression patterns of small secreted proteins.** The transcripts indicated by v1.0 protein ID numbers were significantly up-regulated (B-H P < 0.05 by CyberT) in either maize or tomato (Additional file 3: Table S2). Transcript levels for ID 17705 (ID 230434 in v2.0) and ID 19757 were measured also by qPCR, and the data shown here are also plotted in Figure 3B, where these genes are labelled SSP and HYD2, respectively.

Genes encoding small secreted cysteine-rich proteins (SSPs) in the *T. virens* genome have been annotated based on the following criteria: 300 amino acids or less, at least four cysteine residues, predicted signal peptide for secretion, and lack of annotated enzyme activity [43,60]. Several genes belonging to this (heterogenous) class were up-regulated in interaction with maize or tomato (Additional file 3: Table S2, Figure 8). Up-regulation of transcript 17705 is specific to maize, and this was validated by qPCR (Figure 3B). This gene in v1.0 of the *T. virens* sequence database corresponds to ID 230434 in v2.0; in v1.0 the protein model was incomplete, lacking the N-terminus, and thus was not included in the original SSP list compiled by Kubicek et al. [43]. We added the complete model 230434 to the previous clustering analysis, and found that it belongs to cluster 3, a SSP family of unknown function [60]. ID 19757 is a predicted class II hydrophobin (HfgII), significantly up-regulated in response to tomato roots (Additional file 3: Table S2, Figure 3A), and showing an up-regulated trend in the average transcript abundance in interaction with maize (Figures 3 and 8). A class II hydrophobin was induced in *T. harzianum* by the presence of tomato plants [46]. This protein shows homology to a class II hydrophobin from *T. virens* (best hit by BLASTP search of the v2.0 database: 91466 4.34E-47), related only more distantly to 19757. Both *T. virens* proteins show the same core structure containing eight cysteine residues arranged in a conserved motif. The ceratoplatanin family gene Sm1, encoding a SSP which is an inducer of systemic resistance, was up-regulated in interaction with cotton [61]. Samolski et al. [46] found a 2.3-fold induction in interaction with tomato, while here there was only a 1.2-fold (not significant) increase in response to tomato roots. The cell wall protein QID74 [62] is unfortunately not on the microarray because the *T. virens* ortholog was missing from the v1.0 gene models. Thus, direct comparison with expression of SSP genes in previous studies is not straightforward, probably due to different plant hosts and conditions.

The “cluster 3” SSP (ID 17705 v1.0/230434 v2.0) seems a promising candidate for functional analysis as a maize-interaction-specific small secreted protein.

### Functional significance of regulated genes

The strong induction of genes for enzymes with the potential to degrade plant cell walls is interesting, as the interaction between *T. virens* and these two hosts is considered mutualistic. This finding is consistent with previous reports [46,47]. In the mutualistic interaction of barley with the basidiomycete *Piriformospora indica* –, hydrolytic enzymes and transporters are strongly induced following the transition from biotrophy to saprotrophic growth on dead root cortex cells. There is relatively little induction of these genes in the biotrophic *P. indica*-*Arabidopsis* interaction [41]. While a full meta-analysis is beyond the scope of this study, the similarity between *P. indica* – barley and the *T. virens* interactions studied here suggests that the *T. virens* – root interactions have a limited necrotrophic aspect, like *P. indica* – barley and in contrast to *P. indica* - *Arabidopsis*. If so, cell death should be observed, and there is preliminary evidence for this from scattered propidium iodide staining of plant and fungal nuclei (Figure 4 and data not shown). Furthermore, this similarity between *P. indica* – barley and the two *T. virens* interactions points to parallel evolution of a mutualistic, ISR-promoting, fungal-root interaction distinct from mycorrhizae, in Basidiomycota and Ascomycota.

The genes identified here and in previous studies can provide specific reporters for the *T. virens*-root interaction. To test this concept we constructed a reporter in which GFP was expressed under control of the predicted upstream regulatory region from the 90504 gene. Transgenic lines expressing this construct showed strong GFP fluorescence in interaction with roots (Figure 4) and root sections (data not shown). The protein sequence of 90504, belonging to glycoside hydrolase (GH) family GH7, is nearly identical (three differences in amino acid sequence, all of which are similar) to the *T. virens* T87 gene *CBHI* (accession ACF93800) which is up-regulated in interaction with tomato [47]. In hydroponic cultures, fluorescent hyphae could be detected in the outer root layers, while externally adhering hyphae showed fluorescence in or on some hyphal compartments (Figure 4). We have not confirmed penetration into root cortex cells under the conditions studied here, though GFP-expressing hyphae are closely associated with maize cells (Figure 4). Higher-resolution localization of *T. virens* hyphae in the root cortex will require a line expressing GFP at high, constitutive levels, like the *T. harzianum* line used in a recent study of the role of salicylic acid in limiting penetration into *Arabidopsis* roots [63]. In the *T. harzianum*- tomato root interaction there is evidence for intracellular hyphae or yeast-like cells [35]. If the *T. virens* – root interaction (at 3 days in hydroponic culture) most resembles the late-stage (14 days) *P. indica*- barley interaction, induction of hydrolytic enzymes might reflect saprotrophic on dead root cells. The majority of root cells at three days interaction, however, appear living, since their nuclei do not stain with propidium iodide (Figure 4B). The genome of the arbuscular mycorrhizal fungus *Rhizophagus irregularis* lacks GH families predicted to degrade plant cell wall polysaccharides [64]. In contrast, these are induced in *T. virens* interacting with roots (for example, GH7, GH28, GH61). Two pectate lyase (GH28) genes are up-regulated in ectomycorrhiza, as is a GH61 gene (Suppl. Table 25 in: [56]). Visualization of the time course and location of expression of the GH genes during colonization should help to understand how *T. virens* fits into the biotroph – saprotroph – pathogen continuum.

*Trichoderma* spp*.* interact with both monocots and dicots. Different patterns of gene expression might facilitate this wide (and agriculturally beneficial) host range. In support of this view, we found that a number of genes were expressed specifically in response to either maize or tomato (Additional file 4: Table S3; Figures 6 and 7). One of these is invertase TvInv [27], expressed more than 100-fold higher on maize than on tomato. Several small secreted proteins are expressed preferentially in each interaction. Short-chain oxidoreductases appear to be characteristic of the maize interaction. Identification of the substrates of these predicted proteins, if they indeed have enzyme activity, might help identify their (specific) role in the *Trichoderma*-maize root interaction. In general, there are not enough functional data to fully understand why a particular set of genes is induced in the maize or tomato mutualism. The existence of specific reporters of each interaction, though, demonstrates specific transcriptomic signatures for the interaction with each plant.

## Conclusions

This study provides a better knowledge of the crosstalk of *Trichoderma virens*, an agriculturally relevant biocontrol agent, with plants. Genes differentially expressed during interaction with plant roots encode proteins belonging to several functional classes including enzymes, transporters and small secreted proteins. Among them, glycoside hydrolases and transporters are highlighted by their abundance and suggest an important factor in the metabolism of host cell walls during colonization of the outer root layers. Activity of the *CBH1* promoter was reported by GFP fluorescence in a transgenic *T. virens* line on roots, providing a first spatial view of how the plant re-programs the fungal transcriptome. Host-specific gene expression may contribute to the ability of *T. virens* to colonize the roots of a wide range of plant species. The host-specific gene list obtained here will facilitate functional experiments to test this hypothesis.

### Availability of supporting data

The microarray data sets supporting the results of this article are available in the GEO repository, http://www.ncbi.nlm.nih.gov/geo/query/acc.cgi?acc=GSE64344.

## Additional files

Additional file 1: Table S4. This file lists the sequences of the oligonucleotide probes printed on the *T. virens* microarray. Additional file 2: Table S1. Microarray data from five biological replicates of *T. virens* alone (Tv1-Tv5), three of Tv cocultured with maize roots (M2-M3) and three with tomato roots (T1-T3) are given as a text file. The first column (#name) indicates the protein model ID from the *T. virens* genome (v1.0, http://genome.jgi-psf.org/cgi-bin/searchGM?db=Trive1), which defines a unique oligonucleotide probe on the array. Probes that were printed n times on the array appear in the table as an independent entries (rows). The microarrays were designed from the v1.0 database, which contained 11,655 gene models. If the intron/exon structure and/or the automated annotation have been updated in v2.0 (http://genome.jgi-psf.org/TriviGv29_8_2/TriviGv29_8_2.home.html, 12,421 gene models), the protein ID number in v2.0 is different. In this case, corresponding models for genes of interest were identified in the v2.0 database by BLASTP search with the protein model from v1.0. The alignments were inspected to see that they covered the predicted coding sequence, and the best hit, usually at an E value of 0.0, was taken as the corresponding v2.0 sequence. Additional file 3: Table S2. Annotated list of genes significantly regulated in interaction with maize or tomato. Significantly regulated genes were identified using CyberT (see Methods). The cutoff for significance was an adjusted Benjamini-Hochberg probability of P < 0.05. Column 1 indicates the *T. virens* v1.0 protein ID number for each gene (protein-encoding transcript) model. The corresponding v2.0 ID numbers, found by BLASTP search of v2.0 with the v1.0 sequence, are given in column 2. “Both” indicates genes significantly up-regulated in interaction with maize and tomato; “maize” and “tomato” lists include those identified by CyberT as significantly up-regulated with either plant host, but not both. Additional file 4: Table S3. Annotated list of genes significantly regulated in interaction with maize or tomato (from Additional file 3: Table S2), which also satisfy the criterion of (maize + Tv) significantly different from (tomato + Tv) at an adjusted Benjamini-Hochberg probability of P < 0.05, calculated using CyberT. In the bar graph at the right, the ratios are plotted on a log_10_ scale (horizontal axis), ordered according to the gene list (vertical axis). Additional file 5: Figure S1. GO term analysis for significantly regulated transcripts. The list of genes from Additional file 3: Table S2 (regulated) was analyzed by Blast2GO as described in the Methods. An arbitrarily chosen list of similar size (control) was analyzed in the same way, for comparison. The pie charts indicate the biological process score distribution.

## References

[1] Schuster A, Schmoll M (2010). Biology and biotechnology of Trichoderma. Appl Microbiol Biotechnol.

[2] Howell CR (2003). Mechanisms employed by Trichoderma species in the biological control of plant diseases: the history and evolution of current concepts. Plant Dis.

[3] Harman GE (2011). Multifunctional fungal symbionts: new tools to enhance plant growth and productivity. New Phytol.

[4] Lorito M, Woo SL, Harman GE, Monte E (2010). Translational research on Trichoderma: from ‘omics to the field. Annu Rev Phytopathol.

[5] Hermosa R, Viterbo A, Chet I, Monte E (2012). Plant-beneficial effects of Trichoderma and of its genes. Microbiology.

[6] Contreras-Cornejo H, Ortiz-Castro R, López-Bucio J, Mukherjee PK, Horwitz BA, Singh US, Mukherjee M, Schmoll M (2013). Promotion of plant growth and the induction of systemic defence by Trichoderma: physiology, genetics and gene expression. Trichoderma: Biology and Applications.

[7] Yedidia I, Srivastva AK, Kapulnik Y, Chet I (2001). Effect of *Trichoderma harzianum* on microelement concentrations and increased growth of cucumber plants. Plant Soil.

[8] Tucci M, Ruocco M, De Masi L, De Palma M, Lorito M (2011). The beneficial effect of Trichoderma spp. on tomato is modulated by the plant genotype. Mol Plant Pathol.

[9] Viterbo A, Landau U, Kim S, Chernin L, Chet I (2010). Characterization of ACC deaminase from the biocontrol and plant growth-promoting agent *Trichoderma asperellum* T203. FEMS Microbiol Lett.

[10] Contreras-Cornejo HA, Macias-Rodriguez L, Cortes-Penagos C, Lopez-Bucio J (2009). *Trichoderma virens*, a plant beneficial fungus, enhances biomass production and promotes lateral root growth through an auxin-dependent mechanism in Arabidopsis. Plant Physiol.

[11] Hohmann P, Jones EE, Hill RA, Stewart A (2011). Understanding Trichoderma in the root system of *Pinus radiata*: associations between rhizosphere colonisation and growth promotion for commercially grown seedlings. Fungal Biol.

[12] Bae H, Sicher RC, Kim MS, Kim SH, Strem MD, Melnick RL (2009). The beneficial endophyte *Trichoderma hamatum* isolate DIS 219b promotes growth and delays the onset of the drought response in *Theobroma cacao*. J Exp Bot.

[13] Donoso EP, Bustamante RO, Caru M, Niemeyer HM (2008). Water deficit as a driver of the mutualistic relationship between the fungus *Trichoderma harzianum* and two wheat genotypes. Appl Environ Microbiol.

[14] Mastouri F, Bjorkman T, Harman GE (2012). *Trichoderma harzianum* enhances antioxidant defense of tomato seedlings and resistance to water deficit. Mol Plant Microbe Interact.

[15] Moran-Diez ME, Cardoza RE, Gutierrez S, Monte E, Hermosa R (2010). TvDim1 of *Trichoderma virens* is involved in redox-processes and confers resistance to oxidative stresses. Curr Genet.

[16] Rawat R, Tewari L (2011). Effect of abiotic stress on phosphate solubilization by biocontrol fungus Trichoderma sp. Curr Microbiol.

[17] Djonovic S, Vargas WA, Kolomiets MV, Horndeski M, Wiest A, Kenerley CM (2007). A proteinaceous elicitor Sm1 from the beneficial fungus *Trichoderma virens* is required for induced systemic resistance in maize. Plant Physiol.

[18] Viterbo A, Wiest A, Brotman Y, Chet I, Kenerley C (2007). The 18mer peptaibols from *Trichoderma virens* elicit plant defence responses. Mol Plant Pathol.

[19] Shoresh M, Harman GE, Mastouri F (2010). Induced systemic resistance and plant responses to fungal biocontrol agents. Annu Rev Phytopathol.

[20] Salas-Marina MA, Silva-Flores MA, Cervantes-Badillo MG, Rosales-Saavedra MT, Islas-Osuna MA, Casas-Flores S (2011). The Plant Growth-Promoting Fungus *Aspergillus ustus* Promotes Growth and Induces Resistance Against Different Lifestyle Pathogens in *Arabidopsis thaliana*. J Microbiol Biotechnol.

[21] Yedidia I, Shoresh M, Kerem Z, Benhamou N, Kapulnik Y, Chet I (2003). Concomitant induction of systemic resistance to *Pseudomonas syringae pv. lachrymans* in cucumber by *Trichoderma asperellum* (T-203) and accumulation of phytoalexins. Appl Environ Microbiol.

[22] Yoshioka Y, Ichikawa H, Naznin HA, Kogure A, Hyakumachi M (2012). Systemic resistance induced in *Arabidopsis thaliana* by *Trichoderma asperellum* SKT-1, a microbial pesticide of seedborne diseases of rice. Pest Manag Sci.

[23] Vargas WA, Djonovic S, Sukno SA, Kenerley CM (2008). Dimerization controls the activity of fungal elicitors that trigger systemic resistance in plants. J Biol Chem.

[24] Avis PG, Mueller GM, Lussenhop J (2008). Ectomycorrhizal fungal communities in two North American oak forests respond to nitrogen addition. New Phytol.

[25] Martinez-Medina A, Roldan A, Pascual JA (2011). Interaction between arbuscular mycorrhizal fungi and *Trichoderma harzianum* under conventional and low input fertilizer condition in melon crops: growth response and Fusarium wilt biocontrol. App Soil Ecol.

[26] de Santiago A, Quintero JM, Aviles M, Delgado A (2011). Effect of *Trichoderma asperellum* strain T34 on iron, copper, manganese, and zinc uptake by wheat grown on a calcareous medium. Plant Soil.

[27] Vargas WA, Mandawe JC, Kenerley CM (2009). Plant-derived sucrose is a key element in the symbiotic association between Trichoderma virens and maize plants. Plant Physiol.

[28] Shoresh M, Harman GE (2008). The molecular basis of shoot responses of maize seedlings to *Trichoderma harzianum* T22 inoculation of the root: a proteomic approach. Plant Physiol.

[29] Brotman Y, Landau U, Cuadros-Inostroza A, Tohge T, Fernie AR, Chet I (2013). Trichoderma-plant root colonization: escaping early plant defense responses and activation of the antioxidant machinery for saline stress tolerance. PLoS Pathog.

[30] Hoyos-Carvajal L, Orduz S, Bisset J (2009). Growth stimulation in bean (*Phaseolus vulgaris L.*) by Trichoderma. Biol Contr.

[31] Adams P, De-Leij FA, Lynch JM (2007). *Trichoderma harzianum* Rifai 1295–22 mediates growth promotion of crack willow (Salix fragilis) saplings in both clean and metal-contaminated soil. Microb Ecol.

[32] Babu AG, Shim J, Bang KS, Shea PJ, Oh BT (2014). *Trichoderma virens* PDR-28: a heavy metal-tolerant and plant growth-promoting fungus for remediation and bioenergy crop production on mine tailing soil. J Environ Manage.

[33] Harman GE, Howell CR, Viterbo A, Chet I, Lorito M (2004). Trichoderma species–opportunistic, avirulent plant symbionts. Nat Rev Microbiol.

[34] Yedidia II, Benhamou N, Chet II (1999). Induction of defense responses in cucumber plants (*Cucumis sativus* L. ) By the biocontrol agent *Trichoderma harzianum*. Appl Environ Microbiol.

[35] Chacon MR, Rodriguez-Galan O, Benitez T, Sousa S, Rey M, Llobell A (2007). Microscopic and transcriptome analyses of early colonization of tomato roots by *Trichoderma harzianum*. Int Microbiol.

[36] Martin F, Aerts A, Ahren D, Brun A, Danchin EG, Duchaussoy F (2008). The genome of *Laccaria bicolor* provides insights into mycorrhizal symbiosis. Nature.

[37] Plett JM, Kemppainen M, Kale SD, Kohler A, Legue V, Brun A (2011). A secreted effector protein of *Laccaria bicolor* is required for symbiosis development. Curr Biol.

[38] Plett JM, Martin F (2012). Poplar root exudates contain compounds that induce the expression of MiSSP7 in *Laccaria bicolor*. Plant Signal Behav.

[39] Plett JM, Daguerre Y, Wittulsky S, Vayssieres A, Deveau A, Melton SJ, Kohler A, Morrell-Falvey JL, Brun A, Veneault-Fourrey C (2014). Effector MiSSP7 of the mutualistic fungus *Laccaria bicolor* stabilizes the Populus JAZ6 protein and represses jasmonic acid (JA) responsive genes. Proceedings of the National Academy of Sciences of the United States of America.

[40] Kloppholz S, Kuhn H, Requena N (2011). A secreted fungal effector of *Glomus intraradices* promotes symbiotic biotrophy. Curr Biol.

[41] Lahrmann U, Ding Y, Banhara A, Rath M, Hajirezaei MR, Dohlemann S (2013). Host-related metabolic cues affect colonization strategies of a root endophyte. Proc Natl Acad Sci U S A.

[42] Martinez D, Berka RM, Henrissat B, Saloheimo M, Arvas M, Baker SE (2008). Genome sequencing and analysis of the biomass-degrading fungus *Trichoderma reesei* (syn. *Hypocrea jecorina*). Nat Biotechnol.

[43] Kubicek CP, Herrera-Estrella A, Seidl-Seiboth V, Martinez DA, Druzhinina IS, Thon M (2011). Comparative genome sequence analysis underscores mycoparasitism as the ancestral life style of Trichoderma. Genome Biol.

[44] Druzhinina I, Seidl-Seiboth V, Herrera-Estrella A, Horwitz B, Kenerley C, Monte E (2011). Trichoderma: the genomics of opportunistic success. Nat Rev Microbiol.

[45] Mukherjee PK, Horwitz BA, Herrera-Estrella A, Schmoll M, Kenerley CM (2013). Trichoderma research in the genome era. Annu Rev Phytopathol.

[46] Samolski I, de Luis A, Vizcaino JA, Monte E, Suarez MB (2009). Gene expression analysis of the biocontrol fungus *Trichoderma harzianum* in the presence of tomato plants, chitin, or glucose using a high-density oligonucleotide microarray. BMC Microbiol.

[47] Rubio MB, Dominguez S, Monte E, Hermosa R (2012). Comparative study of Trichoderma gene expression in interactions with tomato plants using high-density oligonucleotide microarrays. Microbiology.

[48] Mehrabi-Koushki M, Rouhani H, Mahdikhani-Moghaddam E (2012). Differential Display of Abundantly Expressed Genes of *Trichoderma harzianum* During Colonization of Tomato-Germinating Seeds and Roots. Curr Microbiol.

[49] Trushina N, Levin M, Mukherjee PK, Horwitz BA (2013). PacC and pH-dependent transcriptome of the mycotrophic fungus *Trichoderma virens*. BMC Genomics.

[50] Hatfield GW, Hung SP, Baldi P (2003). Differential analysis of DNA microarray gene expression data. Mol Microbiol.

[51] Kayala MA, Baldi P (2012). Cyber-T web server: differential analysis of high-throughput data. Nucleic Acids Res.

[52] Sturn A, Quackenbush J, Trajanoski Z (2002). Genesis: cluster analysis of microarray data. Bioinformatics.

[53] Maor R, Puyesky M, Horwitz BA, Sharon A (1998). Use of green fluorescent protein (GFP) for studying development and fungal-plant interaction in *Cochliobolus heterostrophus*. Mycol Res.

[54] Lu S, Lyngholm L, Yang G, Bronson C, Yoder OC, Turgeon BG (1994). Tagged mutations at the Tox1 locus of *Cochliobolus heterostrophus* by restriction enzyme-mediated integration. Proc Natl Acad Sci U S A.

[55] Turgeon B, Condon B, Liu J, Zhang N, Sharon A (2010). Protoplast transformation of filamentous fungi. Molecular and Cell Biology Methods for Fungi.

[56] Martin F, Kohler A, Murat C, Balestrini R, Coutinho PM, Jaillon O (2010). Perigord black truffle genome uncovers evolutionary origins and mechanisms of symbiosis. Nature.

[57] Caffall KH, Mohnen D (2009). The structure, function, and biosynthesis of plant cell wall pectic polysaccharides. Carbohydr Res.

[58] O’Connell RJ, Thon MR, Hacquard S, Amyotte SG, Kleemann J, Torres MF (2012). Lifestyle transitions in plant pathogenic Colletotrichum fungi deciphered by genome and transcriptome analyses. Nat Genet.

[59] Moran-Diez E, Hermosa R, Ambrosino P, Cardoza RE, Gutierrez S, Lorito M (2009). The ThPG1 endopolygalacturonase is required for the *Trichoderma harzianum*-plant beneficial interaction. Mol Plant Microbe Interact.

[60] Horwitz BA, Kosti I, Glaser F, Mukherjee M, Mukherjee PK, Horwitz BA, Singh US, Mukherjee M, Schmoll M (2013). Trichoderma genomes: a vast reservoir of potential elicitor proteins. Trichoderma: Biology and Applications.

[61] Djonovic S, Pozo MJ, Dangott LJ, Howell CR, Kenerley CM (2006). Sm1, a proteinaceous elicitor secreted by the biocontrol fungus *Trichoderma virens* induces plant defense responses and systemic resistance. Mol Plant Microbe Interact.

[62] Samolski I, Rincon AM, Pinzon LM, Viterbo A, Monte E (2012). The qid74 gene from *Trichoderma harzianum* has a role in root architecture and plant biofertilization. Microbiology.

[63] Alonso-Ramírez A, Poveda J, Martin I, Hermosa R, Monte R, Nicolás C (2014). Salicylic acid prevents *Trichoderma harzianum* from entering the vascular system of roots. Mol Plant Pathol.

[64] Tisserant E, Malbreil M, Kuo A, Kohler A, Symeonidi A, Balestrini R (2013). Genome of an arbuscular mycorrhizal fungus provides insight into the oldest plant symbiosis. Proc Natl Acad Sci U S A.

