# Supplementary figures and images for: Host-specific transcriptomic pattern of Trichoderma virens during interaction with maize or tomato roots
Source: BMC Genomics. 2015 Jan 22;16(1):8. doi: 10.1186/s12864-014-1208-3 (PMC4326404; doi:10.1186/s12864-014-1208-3)

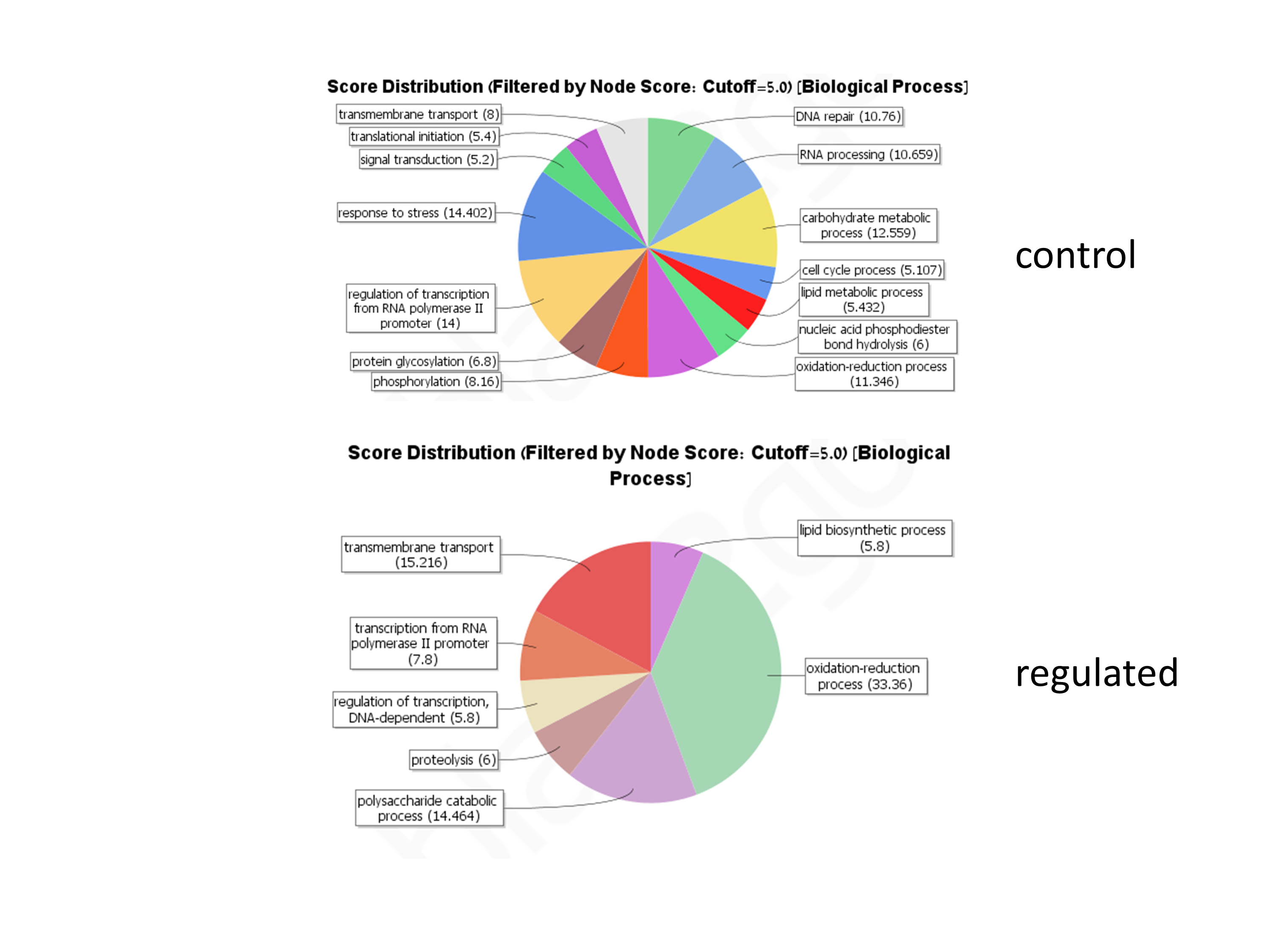

Supplement: Additional file 5: Figure S1. — GO term analysis for significantly regulated transcripts. The list of genes from Additional file 3: Table S2 (regulated) was analyzed by Blast2GO as described in the Methods. An arbitrarily chosen list of similar size (control) was analyzed in the same way, for comparison. The pie charts indicate the biological process score distribution. [file 12864_2014_1208_MOESM5_ESM.tif]
